# A Real-Time Embedded System for Driver Drowsiness Detection Based on Visual Analysis of the Eyes and Mouth Using Convolutional Neural Network and Mouth Aspect Ratio

**DOI:** 10.3390/s24196261

**Published:** 2024-09-27

**Authors:** Ruben Florez, Facundo Palomino-Quispe, Ana Beatriz Alvarez, Roger Jesus Coaquira-Castillo, Julio Cesar Herrera-Levano

**Affiliations:** 1 LIECAR Laboratory, Universidad Nacional de San Antonio Abad del Cusco (UNSAAC), Cusco 08003, Peru; facundo.palomino@unsaac.edu.pe (F.P.-Q.); roger.coaquira@unsaac.edu.pe (R.J.C.-C.); julio.herrera@unsaac.edu.pe (J.C.H.-L.); 2 PAVIC Laboratory, University of Acre (UFAC), Rio Branco 69915-900, Brazil; ana.alvarez@ufac.br

**Keywords:** convolutional neural network (CNN), driver monitoring system, drowsiness detection, mouth aspect ratio (MAR), NVIDIA Jetson Nano, yawning detection

## Abstract

Currently, the number of vehicles in circulation continues to increase steadily, leading to a parallel increase in vehicular accidents. Among the many causes of these accidents, human factors such as driver drowsiness play a fundamental role. In this context, one solution to address the challenge of drowsiness detection is to anticipate drowsiness by alerting drivers in a timely and effective manner. Thus, this paper presents a Convolutional Neural Network (CNN)-based approach for drowsiness detection by analyzing the eye region and Mouth Aspect Ratio (MAR) for yawning detection. As part of this approach, endpoint delineation is optimized for extraction of the region of interest (ROI) around the eyes. An NVIDIA Jetson Nano-based device and near-infrared (NIR) camera are used for real-time applications. A Driver Drowsiness Artificial Intelligence (DD-AI) architecture is proposed for the eye state detection procedure. In a performance analysis, the results of the proposed approach were compared with architectures based on InceptionV3, VGG16, and ResNet50V2. Night-Time Yawning–Microsleep–Eyeblink–Driver Distraction (NITYMED) was used for training, validation, and testing of the architectures. The proposed DD-AI network achieved an accuracy of 99.88% with the NITYMED test data, proving superior to the other networks. In the hardware implementation, tests were conducted in a real environment, resulting in 96.55% and 14 fps on average for the DD-AI network, thereby confirming its superior performance.

## 1. Introduction

According to the World Health Organization (WHO), “Every year the lives of approximately 1.3 million people are cut short as a result of a road traffic crash. Between 20 and 50 million more people suffer non-fatal injuries, with many incurring a disability as a result of their injury”. It is estimated that 13 million more people will die and 500 million more will be injured in the next decade, especially in low-income and middle-income countries. These numbers are not acceptable in either absolute or relative terms. Vehicle accidents are the leading cause of death worldwide, despite the fact that all such deaths and injuries are preventable [1].

**Figure 1:**
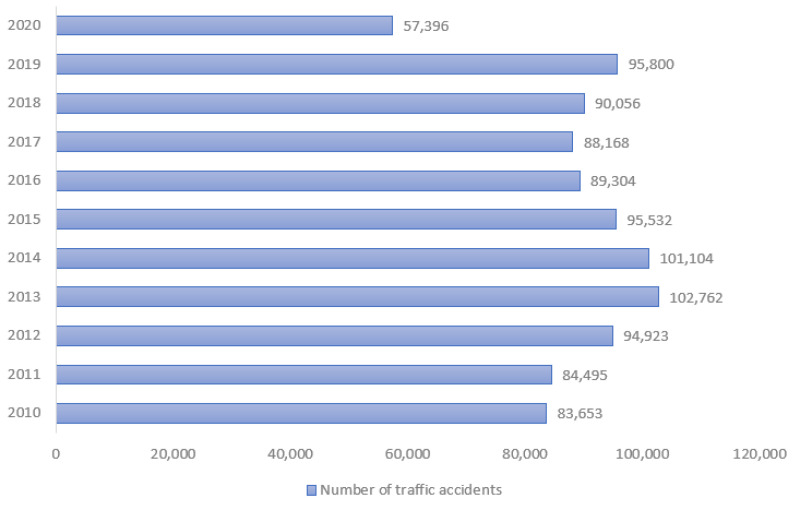
Traffic accident trend 2010–2020 in Perú. Report by PNP.

In Peru, the statistics of the National Police of Peru (PNP) show that 983,193 accidents were registered during the last decade (2010–2020), as shown in Figure 1. The year with the highest number of road accidents was 2013 (102,762), followed by 2014 (101,104) and 2019 (95,800) [2]. In the first semester of the year 2022, 38.53% of traffic accident injuries occurred in Lima, while 61.47% occurred in the rest of Peru [3].

The factors that contributed to the occurrence of vehicular accidents were human factors, vehicle factors, infrastructure factors, road environment factors, and undetectable or unclear causes. These are listed in Table 1 along with their respective indices from 2019 to 2022. The human factor concentrates on the causes of road accidents, varying from 75.5% (35,959) in 2019 to 70.4% (29,186) in 2022. In this sense, regarding human factors it was shown that the driver was the main cause of road accidents, with 93.9% (27396) of road accidents in Peru caused by drivers in 2022 [4].

It was found that the causes within the human factor with regard to drivers are speeding, driver drunkenness, driver recklessness, and driver drowsiness. Looking at the above causes, it is not difficult to deduce their connection with sleep and drowsiness, as drivers tend to drive faster when tired due to wanting to reach the destination quickly. Similarly, a drunk driver is more likely to fall asleep at the wheel, and a reckless driver usually chooses to drive despite being tired. This was observed in a publication by the Ministry of Health, which showed that 30% of accidents in Peru were related to tiredness and drowsiness at the wheel [5].

Drowsiness while driving is a state of tiredness and exhaustion caused by a lack of sleep, fatigue, alcohol consumption the day before, overeating before driving, or overexertion. These and other factors inhibit the body’s ability to respond, which can lead to tragedy or traffic accidents.

**Figure 2:**
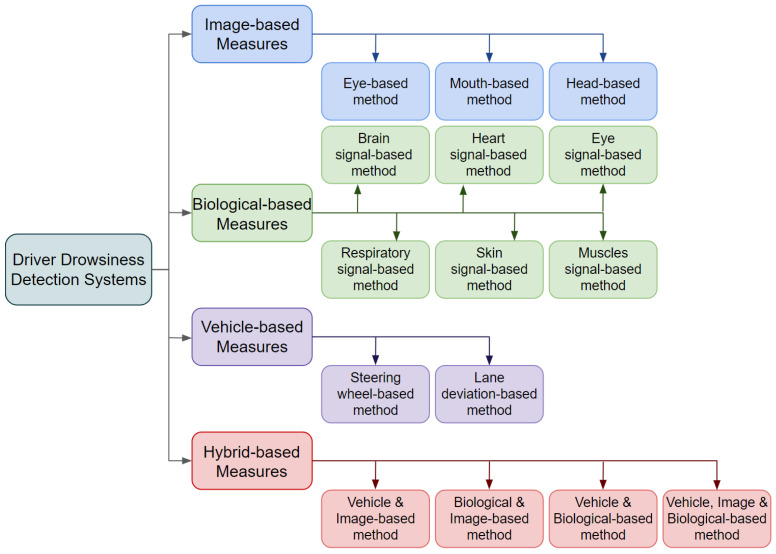
Driver drowsiness detection measures. Source by [6].

Currently, there are many related studies on the detection of drowsiness that contemplate different methods and techniques. A study by Albadawi et al. [6] states that to determine the different stages of drowsiness, they studied drivers’ reactions and driving habits. The graph in Figure 2 shows all measures currently used to classify driver drowsiness. Two types measures, namely, imaging and biometric data, involve observing the driver. The third type, vehicle-based measures, are taken from the car itself. The fourth type consists of mixed measures that combine at least two of the other types.

Most current studies focus on image-based measures, as signs of drowsiness are often visible and can be recorded by cameras or visual sensors. These include facial expressions and driver movements, especially of the head and eyes, as well as mouth movements. This method is very reliable; its main feature is that it is non-invasive, and as such does not cause harm or discomfort to the driver.

Most image-based systems rely on deep learning techniques such as Convolutional Neural Networks [7,8,9,10] for the extraction of features from images. Such approaches include digital image processing and computer vision. A GPU must be used to ensure correct operation, as these techniques require high computational power.

For implementation in vehicles, an embedded system that satisfies the computational requirements is required to perform the correct operation of the aforementioned techniques.

For this reason, there is a need to develop a portable, robust, and easy to use in-vehicle system that detects and warns drivers of the onset of drowsiness to prevent road accidents. By using convolutional neural network techniques and real-time computer vision, the system does not need to obstruct the driver’s visibility. Several proposals to meet this need can be found in the literature [11,12,13,14,15,16,17,18,19], as described in detail in Section 2.

In this study, a portable real-time constant monitoring system for detecting drowsiness in drivers is proposed. This system is based on convolutional neural network algorithms such as InceptionV3 [20], VGG16 [21], ResNet50V2 [22], and a proposed network for learning the state of the eyes (open or closed) and Mouth Aspect Ratio (MAR) [23] for yawning detection by extracting facial points through Mediapipe [24]. The system is composed of an NVIDIA Jetson Nano, which is the component where all the processing and programming logic is executed; an RGB-NIR camera in charge of obtaining the images in sequence (video) of the driver; a 7-inch screen, which is the graphic interface where the driver’s status is visualized in real time; and a pair of horns which produce sound alarms when drowsiness is detected to warn the driver and avoid an accident.

This research makes the following contributions:

1. An improved technique is proposed for extracting the area of interest (ROI) corresponding to the eyes using MediaPipe, correcting the area created by detecting points on the face, and guaranteeing the ROI of the eyes in the different head postures performed by the driver.
2. A Convolutional Neural Network (CNN)-based approach for drowsiness detection using the Mouth Aspect Ratio (MAR) is proposed, where the CNN is used to perform transfer learning training for feature extraction and eye state learning based on the InceptionV3, VGG16, ResNet50V2 networks. Another CNN network named “DD-AI” is proposed and trained from start to finish to detect yawning using MAR by extracting the facial points of the mouth.
3. An evaluation of the four networks using the Grad-CAM technique to observe the inference performance of each network when classifying the eye state images and obtaining the heat maps of each network, making it possible to visualize the regions that have greater importance in each CNN model.
4. A comparison and discussion of experimental results obtained in other studies using the same approach, allowing for observation of the overall system performance, precision, and accuracy.
5. A portable, robust, efficient, fast, and low-cost system for detecting drowsiness in real time.

The current research represents a significant improvement over the previously presented research in [25] by optimizing the ROI selection method, incorporating an Near-Infrared (NIR) camera, and implementing an embedded system (hardware) based on the methodology of [26].

The rest of this article is organized as follows: Section 2 discusses previous research work related to drowsiness detection systems; Section 3 presents the methodology of the proposed system; Section 4 describes the materials and methods used in the proposed methodology, focusing on the preliminary results of implementing the CNN architecture types; Section 5 describes the implementation of the embedded system; Section 6 presents the experimental results obtained in a real driving environment to demonstrate the proposed system’s performance; Section 7 discusses the results and provides a comparison with other systems; finally, Section 8, concludes the paper and lays out future work.

## 2. Related Work

This section describes previous studies related to detecting drowsiness in drivers in which priority was given to the use of deep learning through CNN architectures and implementation in an embedded system such as a cell phone, Raspberry Pi, or NVIDIA Jetson.

In the system presented by Reddy et al. [11], three CNN architectures were proposed. “Baseline-4” has four inputs (eyes, mouth, and face), while “Baseline-2” has two inputs (left eye and mouth); both are classified into three outputs (normal, yawning, drowsy) and trained on a computer with GPU 1080 GTX and CPU i7-2600. The third, “Compressed-2”, is a compressed version of “Baseline-2” and was implemented on the NVIDIA Jetson TK1 embedded system, where it achieved 89.5% accuracy, 18.9 ms detection time, and 14.9 fps.

Jabbar et al. [12] proposed a multilayer perceptron architecture model with three hidden layers for binary classification (drowsiness and non drowsiness) using the NTHU database [27]. For extraction of the facial points, they made use of Dlib [28], training their neural network on the facial image. The training used an Intel Core i7-7500U computer with a GTX 1080 GPU and resulted in an accuracy of 81% in validation running at 43.4 ms with 7.0 fps. The authors indicated that their neural network model can be integrated into embedded systems such as an Android cell phone. Their main objective was to develop an algorithm that adapts to embedded systems by reducing the size of the neural network model, ultimately obtaining a model size of 100 KB.

Jabbar et al. [13] later improved the architecture of the multilayer perceptron model described above using a convolutional neural network. The training was performed on an Intel Xeon Gold 6128 computer with an NVIDIA QUADRO P4000 GPU, obtaining an accuracy of 83.3% in validation. The CNN model was then reduced to a maximum size of 75 KB to adapt to a Samsung Galaxy S8 Plus embedded system, obtaining execution results of 142 ms and 234.25 fps.

He et al. [14] designed a two-stage CNN, with one network used for eye and mouth detection and localization and the other for normal, warning, and danger classification. The YawDDD [29] dataset was used in combination with their own dataset. Training was conducted on an i5-8300H computer with a GTX 1060 GPU. In the preliminary results, they obtained an accuracy of 93.83% in validation. Their model was added to a Raspberry Pi 4 embedded system, where they obtained an execution time of 96.3 ms at 10.4 fps with a drowsiness detection accuracy of 94.7%.

Çıvık and Yüzgeç [15] used the VGG16 CNN in two models, one to classify the eye state and the other to classify the mouth state. They used Dlib for extraction of the facial points, training with the YawDDD dataset on an i7-7500 computer with an Nvidia GeForce 950M GPU. They used the NVIDIA Jetson Nano embedded system to deploy their model, obtaining an average accuracy of 94.05% at 6 fps.

The system proposed by Li et al. [16] includes a CNN network called “LittleFace” that classifies faces into “normal” and “distracted”. This network was trained with the AFLW dataset [30] to detect drowsiness using the Eye Aspect Ratio (EAR) [31]. The Mouth Aspect Ratio (MAR) was used for the mouth state and a proprietary network was used for facial point detection. They implemented their model on the NVIDIA Jetson Nano embedded system and obtained an average accuracy of 89.55% for drowsiness detection at 58 fps.

Rahman et al. [17] presented a complete system for drowsiness detection using the Eye Aspect Ratio (EAR) and Mouth Aspect Ratio (MAR) to detect drowsiness, with Dlib (ref-dlib) used to extract facial points. They used their CNN architecture only to classify whether the driver was wearing a mask, not for drowsiness detection. They used an AD8232 sensor with an Arduino to monitor the heart rate to ensure that the system is able to monitor the driver’s state at all times. Their entire drowsiness detection system is controlled by the NVIDIA Jetson Nano embedded system, achieving an accuracy of 97.44% on their drowsiness detection tests.

The system called “SOMN_IA” proposed by Flores-Monroy et al. [18] makes use of a proposed CNN architecture called “S-CNN”. The system has three outputs (open eye, closed eye, and distraction). The authors used the NTHU-DDD dataset [27] to train their model and Mediapipe for extraction of facial points. The system focuses only on the state of the eyes, ignoring the mouth state (i.e., yawning). The implementation was performed on an embedded system, but the authors did not indicate which one. However, from the power characteristics and implementation images it can be deduced that it was an NVIDIA Jetson Nano. The authors obtained 95.77% overall accuracy for drowsiness detection under different conditions, with the system working at 21 fps.

N.T. Singh et al. [19] proposed a drowsiness detection system focused on the eye region. Their system uses two CNNs based on Inception-v3 and DenseNet with transfer learning. Although they did not specify the microcontroller used for testing, they achieved 98.1% accuracy with the Inception-v3 network. The system triggers an alert when the driver’s eyes remain closed for 5 to 8 s.

Most related studies [11,12,13,14,15,16,17,19] have the disadvantage of using an RGB camera, which significantly limits drowsiness detection in nighttime environments and varied lighting conditions. In addition, a number of prior studies [12,13,18,19] do not address yawning detection. These issues are discussed further in Section 7.

The present research proposes a hybrid CNN-based approach that uses the MAR to detect drowsiness in drivers. We propose a method for ROI correction in the eye area for binary classification (drowsiness and non-drowsiness) using a CNN evaluated on four CNN architectures: three using transfer learning and a fourth trained from scratch.

## 3. Proposed Methodology

**Figure 3:**
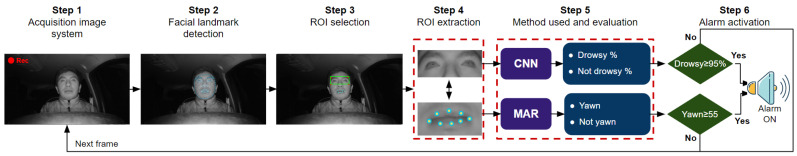
Proposed hybrid system for drowsiness detection.

This chapter describes the proposed hybrid methodology (Figure 3) for real-time detection of driver drowsiness. The proposed system includes five steps, which are described below.

In Step 1, image acquisition is performed to obtain the input data of the system. For this purpose, images are captured with a high-resolution and high-frequency acquisition and infrared illumination (NIR) camera [32]. Regardless of the presence or absence of light, the infrared illumination of the camera will always be active for constant visualization of the driver’s face focusing on the eyes and mouth.

In Step 2, facial landmark detection is performed to detect the points of the face by means of MediaPipe Face Mesh [33], which is a solution that calculates 468 3D facial landmarks in real time using machine learning. Eight points refer to the mouth and four points to the ends of the eyes, which are of interest in Step 3.

After detecting the facial points, Step 3 selects the region of interest (ROI), which is divided into two parts. For the first ROI, corresponding to the area between the eyebrows and above the tip of the nose, Mediapipe Face Mesh provides the following points: 63, 117, 293, 346. A technique for correcting the surrounding area is proposed to ensure that the information of the eye area is not lost when the driver makes lateral movements and head inclination. The second ROI corresponds to the mouth area, which uses the following eight points: 11, 16, 61, 73, 180, 291, 303, 404.

Step 4 involves extraction of the ROIs are extracted. For the eye region, a correction technique is proposed for the surrounding area to ensure that the information of the eye area is not lost when the driver performs lateral movements or head inclination. For the mouth region, the positions of the eight points are extracted to calculate the mouth opening based on the respective distances of those points.

In Step 5, classification of the eye area is carried out by a CNN, applying the CNN architectures of InceptionV3, VGG16, and ResNet50V2 via transfer learning. We propose an additional CNN architecture called “Drowsiness Detection-AI (DD-AI)”, in which four CNNs conduct binary classification of the eye region as either drowsy or not drowsy. The second ROI corresponding to the mouth points is used to classify yawns using the Mouth Aspect Ratio (MAR). A set threshold is used to determine whether or not the driver is yawning. Step 5 involves a hybrid of the CNN-based and MAR-based approaches used for the eye and mouth regions, respectively.

Finally, using the results obtained from Step 5 classification, an audible alarm is activated in Step 6 to warn the driver in the event that symptoms of drowsiness are detected based on the state of the eyes and mouth. In addition, the driver’s status can be viewed in real time on a 7-inch display, which is important to allow the driver or passenger(s) to monitor driver drowsiness.

## 4. Materials and Methods

This section describes the materials and methods used to construct the proposed methodology. Step 5 is discussed in depth, as it contains the proposed hybrid method utilizing CNN and MAR. The four CNN architectures were trained on a computer with an AMD Ryzen 7 5800H CPU, NVIDIA GeForce RTX 3060 6 GB GPU, and 16 GB RAM. The preliminary results of the four CNN architectures and the choice of the best CNN for implementation of the system presented in Section 5 is evaluated as well.

### 4.1. Step 1: Image Acquisition System

Data acquisition is provided by an NIR camera installed on the dashboard of the car in front of the driver and behind the steering wheel. The camera captures live videos of the driver under different light conditions (day, dusk, night) focusing on a single face, corresponding to the driver’s face, which is the main target to be analyzed.

### 4.2. Step 2: Facial Landmark Detection

**Figure 4:**
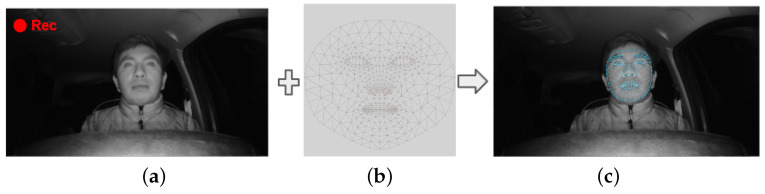
Facial landmark detection using MediaPipe: (**a**) input image, (**b**) MediaPipe face mesh mask, and (**c**) facial landmark detection in an image.

Facial landmark detection is performed using MediaPipe Face Mesh, which delivers 468 facial landmarks (Figure 4), superimposing the mask of the landmarks on each frame of the input video. From these points, only those needed for the eyes and mouth are chosen.

### 4.3. Step 3: ROI Selection

**Figure 5:**
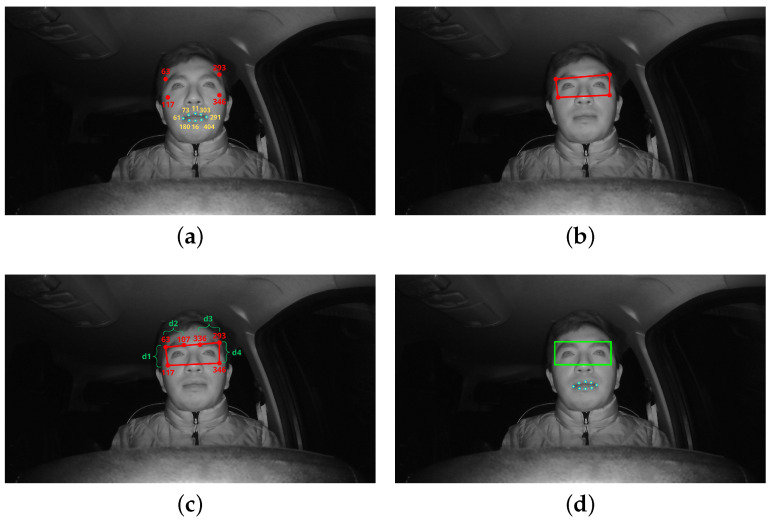
Eye area ROI improvement procedure: (**a**) points for two regions of interest, (**b**) asymmetric eye region of interest, (**c**) region of interest correction method, and (**d**) corrected eye ROI and mouth ROI.

After obtaining the 468 points of the face, four points (63, 117, 293, 346) are chosen for the eye area and eight (11, 16, 61, 73, 180, 291, 303, 404) for to the lips. These points are taken from the mask provided by MediaPipe Face Mesh (Figure 4b), from which the selection of the Region of Interest (ROI) area around the eyes is made, while from the lips only the positions of the points in terms of the “x” and “y” components are extracted. Figure 5 shows the procedure of the proposed method for enhancing the ROI around the eyes. The aforementioned points are selected from the eye and mouth regions and placed on the face, as shown in Figure 5a. Joining the four points of the eye region results in an asymmetric region, as shown in Figure 5b, which makes the process of extracting the ROI difficult.

To solve this problem, we propose an improved version of the technique in [25] to correct this area by comparing distances. First, we take the “x” and “y” components of the right upper extreme points of each eye, in which a new point is placed for the left eye; these points are 63 and 336 for the right and left eyes, respectively. Then, the distances of the extreme and superior points of each eye are found; for the right eye, these are the distance from point 63 to point 117 (d1) and distance from point 63 to point 107 (d2), resulting in a new point being placed for this eye, which is 107. For the left eye, we use the distance from point 336 to point 293 (d3) and the distance from point 293 to point 346 (d4). When all these values are found, it is possible to compare the position of the right upper extreme points of each eye in its two components, as shown in Figure 5c.

To finally draw the corrected rectangle of the eye region area, it is important to ensure that the ROI information of the eyes is not lost and that the ROI is always available for further classification irrespective of the position of the driver’s head (e.g., in the presence of left and right rotational movements and tilt movements). Figure 5d shows the corrected ROI obtained using the proposed method. The pseudocode of Algorithm 1 used for ROI correction is shown below to aid in understanding the proposed method.
**Algorithm 1** ROI Correction1:**Input:** Points: [63,117,293,346,107,336]2:**Output:** Corrected_ROI3:$x_{a},y_{a}\leftarrow P_{336}\left[x\right],P_{336}\left[y\right]$4:$x_{b},y_{b}\leftarrow P_{63}\left[x\right],P_{63}\left[y\right]$5:$d_{1}\leftarrow \mathrm{int}(\mathrm{distance}\left(P_{63},P_{117}\right))$6:$d_{2}\leftarrow \mathrm{int}(\mathrm{distance}\left(P_{63},P_{107}\right))$7:$d_{3}\leftarrow \mathrm{int}(\mathrm{distance}\left(P_{336},P_{293}\right))$8:$d_{4}\leftarrow \mathrm{int}(\mathrm{distance}\left(P_{293},P_{346}\right))$9:**if** $x_{a}>x_{b}$ **then**10:    $start\_x,end\_x\leftarrow x_{b},\left(x_{a}+d_{3}\right)$11:**else**12:    $start\_x,end\_x\leftarrow x_{a},\left(x_{b}+d_{2}\right)$13:**end if**14:**if** $y_{a}>y_{b}$ **then**15:    $start\_y,end\_y\leftarrow y_{b},\left(y_{a}+d_{4}\right)$16:**else**17:    $start\_y,end\_y\leftarrow y_{a},\left(y_{b}+d_{1}\right)$18:**end if**19:**if** (end_x−start_x)>10 and (end_y−start_y)<400 **then**20:    start_x,start_y←start_x−10,start_y−1021:    end_x,end_y←end_x+10,end_y+1022:    **Corrected_ROI ← [start_y:end_y, start_x:end_x]**23:**end if**

**Figure 6:**
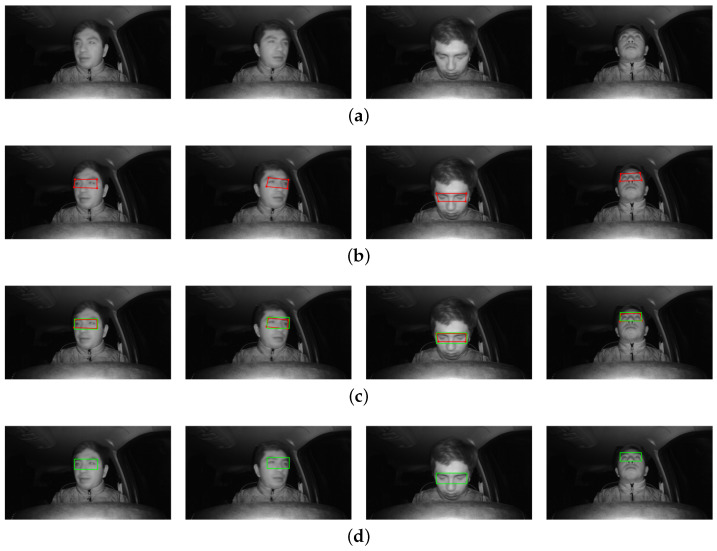
Corrected eye ROIs at different driver head postures: (**a**) head position to the right, (**b**) head position to the left, (**c**) head position down, and (**d**) head position up.

The application of the corrected ROI is illustrated in Figure 6, where different common scenarios for the driver’s head posture are shown.

### 4.4. Step 4: ROI Extraction

**Figure 7:**
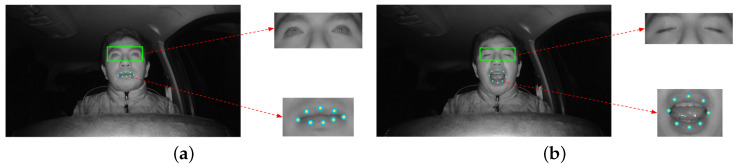
ROIs of the eyes and mouth used to determine the driver’s condition: (**a**) ROI image with open eyes and ROI points with closed mouth; (**b**) ROI image with closed eyes and ROI points with open mouth.

Following correction of the eye area ROI, in this step the previously extracted ROIs of the eye and mouth areas form the inputs of the CNN that makes the classification to determine the drowsiness of the driver. We have two ROIs; the one for the eyes includes two possible states (eyes open or eyes closed), respectively indicating visual drowsiness or no visual drowsiness. The mouth ROI uses eight points to determine whether the driver is yawning (mouth closed or mouth open). Figure 7 shows the ROIs of each region, showing the eight points extracted from the mouth ROI and the image extracted from the eye area ROI.

### 4.5. Step 5: Method and Evaluation

As shown in Figure 3, this step represents the proposed hybrid method, which is divided into two methods for each ROI. In the case of the mouth ROI, the MAR is used based on the eight extracted points. A CNN is used for the eye ROI; the following subsections describe the creation of the dataset, the training experiments considering ten training rounds for each CNN network, and the preliminary results of the training experiments.

#### 4.5.1. Mouth Aspect Ratio (MAR) Method

**Figure 8:**
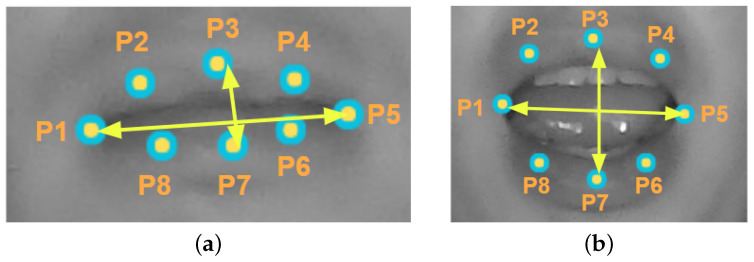
ROIs of the mouth used to determine the driver’s condition. (**a**) Closed Mouth ROI; (**b**) Open Mouth ROI.

The MAR method is used to determine whether or not the driver is yawning. This method uses the Euclidean distance between two points. Sikander and Anwar [23] proposed a method in which they used eight mouth reference points to determine the MAR mouth aspect ratio (Equation (1)) between the height and width of the mouth. These points are shown in Figure 8.

(1) $$MAR=\frac{\left|\right|P_{2}-P_{8}\left|\right|+\left|\right|P_{3}-P_{7}\left|\right|+\left|\right|P_{4}-P_{6}\left|\right|}{3||P_{5}-P_{1}\left|\right|}$$

#### 4.5.2. Convolutional Neural Network (CNN) Method

**Figure 9:**
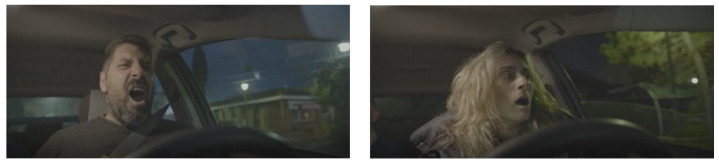
NITYMED dataset. Source by [34].

In order to detect drowsiness by means of the state of the driver’s eyes, it is necessary to use an appropriate dataset. Taking this into account, we considered the Night-Time Yawning–Microsleep–Eyeblink–Driver Distraction (NITYMED) dataset [34] (Figure 9). Compared to the similar datasets [27,29,30], NITYMED presents real case videos of people driving cars while experiencing drowsiness and yawning. The dataset includes people with glasses and different eye sizes as well as both male and female drivers. The dataset contains two classes, namely, “Yawning” and “Microsleep”. The dataset was recorded in RGB format at 25 fps at two different resolutions (1080p and 720p).

From this dataset, we worked with the folder “Full” (1080p) containing the “microsleep” class for “female” (eleven videos) and “male” (twelve videos). From these, we chose six videos (two female and four male): P1043106_na, P1043129_na, P1042751_na, P1042767_na, P1042792_na and P1043075_na. We extracted each frame from these videos.

**Figure 10:**
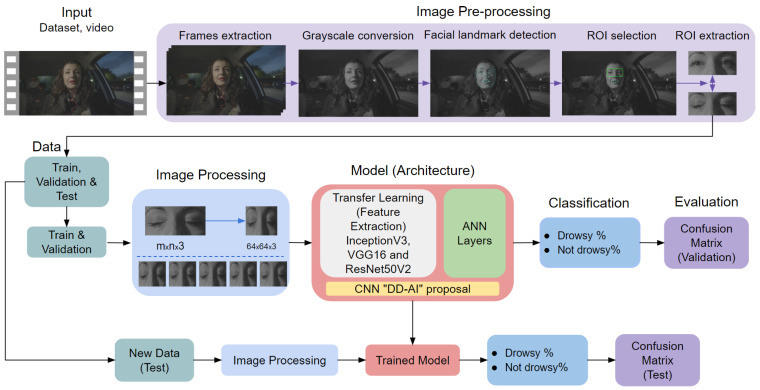
Training procedure using CNN.

Figure 10 shows the method used by the CNNs. In this case, we are dealing with the dataset as input; we then perform the same steps seen in Figure 3 except that two steps, frame extraction and grayscale conversion, are added after recording the videos. During frame extraction, each frame is extracted as an image of the six chosen videos; then, as mentioned above, we work with an NIR camera (three layers). As the dataset needs to be similar to infrared conditions, we converted the images to grayscale while maintaining the three depth layers. Subsequently, the same steps of facial landmark detection, ROI selection, and ROI extraction were performed.

- a. Dataset Creation

**Figure 11:**
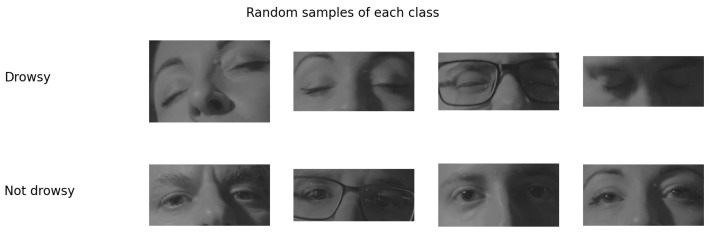
Random samples from each class.

The ROI of the eye area was extracted from the six chosen videos, with the resulting dataset used train the CNNs architectures with 6800 images in total distributed into two classes (drowsy and not drowsy). The drowsy class contained 3400 images and the not drowsy class contained 3400 images. The distribution of the dataset was 70% (4760) for the training set, 15% (1020) for the validation set, and 15% (1020) for the test set. As described, the dataset was balanced for binary classification of eye status. The dataset is summarized in Table 2, with random samples shown in Figure 11.

- b. CNN Training Experiments

At this point, training was performed for each of the four CNNs with ten training experiments for each CNN, resulting in a total of 40 experiments for one image input size. During training, the first training set was used to train the model and the second validation set was used to validate the training. The first step in training the CNNs was to convert image sizes of the training sets. As shown in Figure 11, the images come in different sizes, while for the CNNs they have standard input sizes. In this case, the experiments were performed at a size of 64 × 64 pixels in the case of VGG16, ResNet50V2, and DD-AI and at 75 × 75 pixels in the case of InceptionV3, with all architectures using three depth layers due to the faster processing with the use of the embedded system.

Next, normalization of each image was carried out, consisting of dividing each pixel value of the image by the maximum pixel value. Generally, RGB images come with 8 bits for each pixel; thud, the maximum value of a pixel is 256. However, when working with Python language the initial value is zero; thus, the maximum value of a pixel is 255. To normalize the images, it is necessary to divide each pixel value by the maximum, which is 255, normalizing it from a range of values from 0–255 to a range of values from 0–1. This step was performed to save computational resources.

Another important step is data augmentation, which is performed to avoid overfitting when training CNNs. In this step, five artificial images were created for each image of the training set only. The following parameters were used for data augmentation: rotation_range = 20, horizontal_flip = True, and fill_mode = ’nearest’.

**Figure 12:**
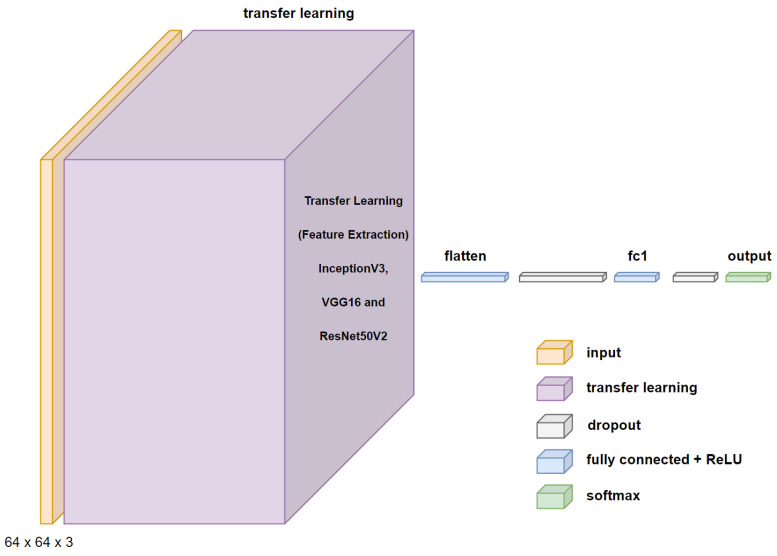
Proposed CNN architecture using transfer learning.

After processing the images, the architectures of each CNN network were created. The first three CNNs used transfer learning, maintaining the weights of the convolutional layers and designing only the architectures of the connected neural networks that perform the binary classification. The design is provided by the flattened layer, followed by a 25% dropout, a dense layer with 500 neurons and ReLU activation, a 25% dropout, and an output layer with two neurons with SoftMax activation. Figure 12 shows the proposed architecture using transfer learning for the InceptionV3, VGG16, and ResNet50V2 networks.

- b.1. Proposed CNN “DD-AI” architecture

**Figure 13:**
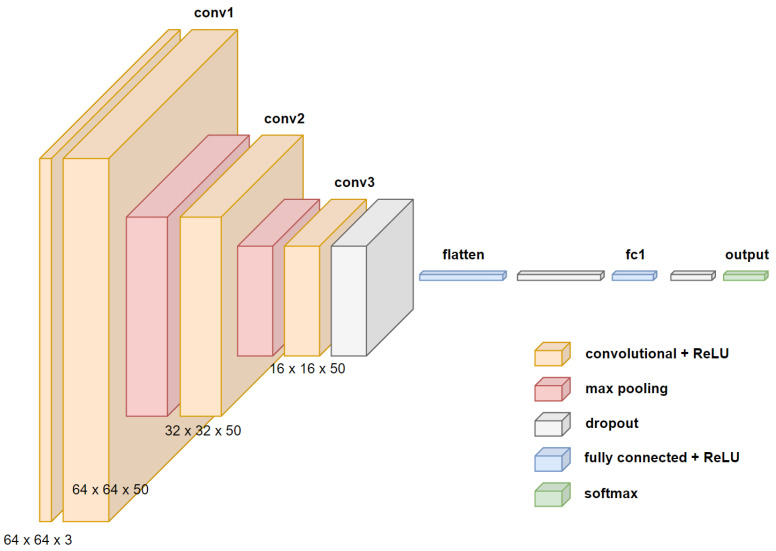
Proposed “DD-AI” CNN architecture.

Figure 13 shows the proposed architecture for drowsiness detection in binary classification. The proposed network consists of the input layer of the images; three convolution layers, each 50 with kernels and ReLU activation; two 2 by 2 max_pooling layers; and a dropout layer with 0.8 between the last convolution layer and the flattened layer. For the classification stage, the same architecture is used with a dense layer with 500 neurons and ReLU activation, two dropout layers with 0.25, and a dense output layer with two neurons and softmax activation.

After designing the four architectures, we proceeded to perform training with each CNN using the parameters listed in Table 3.

- c. Preliminary CNN training results

**Figure 14:**
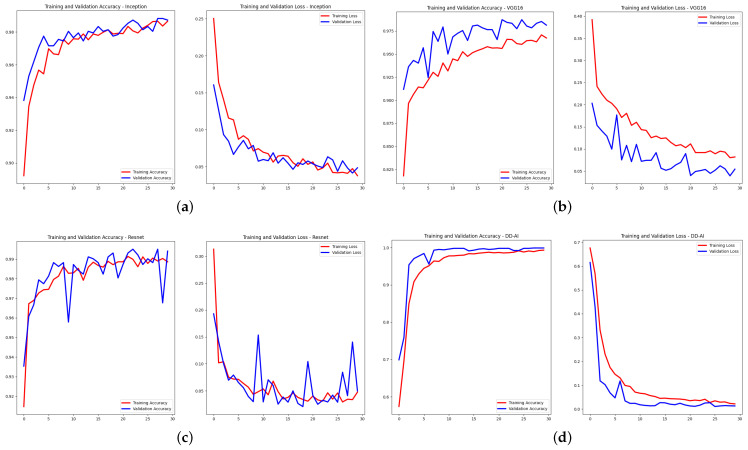
Accuracy and loss graphs of training and validation: (**a**) InceptionV3, (**b**) VGG16, (**c**) ResNet50V2, and (**d**) proposed “DD-AI”.

After performing the training experiments, where each CNN network was trained ten times, we obtained the results shown in Figure 14, presented in terms of accuracy and loss. Out of the ten experiments, the results of the seventh experiment was randomly chosen for each of the four CNN networks.

As can be seen from Figure 14, the proposed “DD-AI” network shows a better response than the other three networks in both training and validation (Figure 14d).

**Figure 15:**
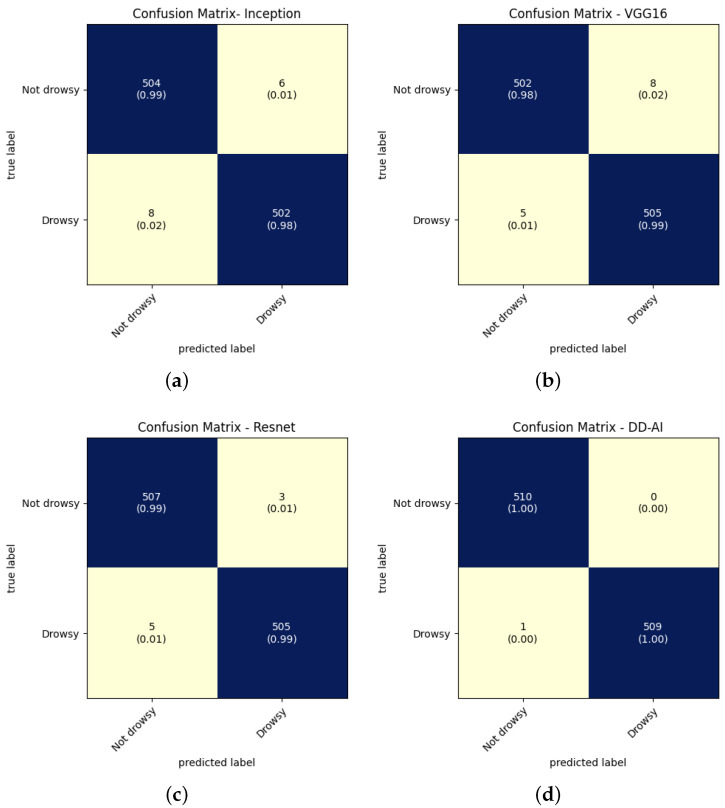
Confusion matrices of CNN validation: (**a**) InceptionV3, (**b**) VGG16, (**c**) ResNet50V2, and (**d**) proposed “DD-AI”.

The results in Figure 15 show the confusion matrices for each CNN for the seventh experiment conducted for each network. These confusion matrices provide four evaluation metrics that serve to determine the performance of the trained CNN classification models. As presented by Caelen [35], these evaluation metrics can be obtained from the confusion matrix by using the following equations:

(2) $$precision=\frac{TP}{TP+FP}$$

(3) $$recall=\frac{TP}{TP+FN}$$

(4) $$F1-score=\frac{2*precision*recall}{precision+recall}$$

(5) $$accuracy=\frac{TP+TN}{TP+TN+FP+FN}$$

where *TP* is true positive, *FP* is false positive, *TN* is true negative, and *FN* is false negative.

The results of the ten training experiments are shown in Table 4, where the calculation of the average and standard deviation in each metric of the four trained CNN is shown. Equations (2)–(5) are used to find the four evaluation metrics discussed above.

Analyzing the results of Table 4, the proposed DD-AI network exceeds an accuracy of 99.84% ± 0.1% for the CNN networks of ResNet50V2, InceptionV3, and VGG16 by 99.39% ± 0.2%, 98.70% ± 0.2%, and 98.57% ± 0.2% respectively. For this particular case, it is necessary to decrease the false negatives of the elementary class (drowsy); thus, the recall has to reach a high value. The DD-AI network has a recall of 99.80%i for the drowsy class with a standard deviation of 0.0%, surpassing the other three networks.

**Figure 16:**
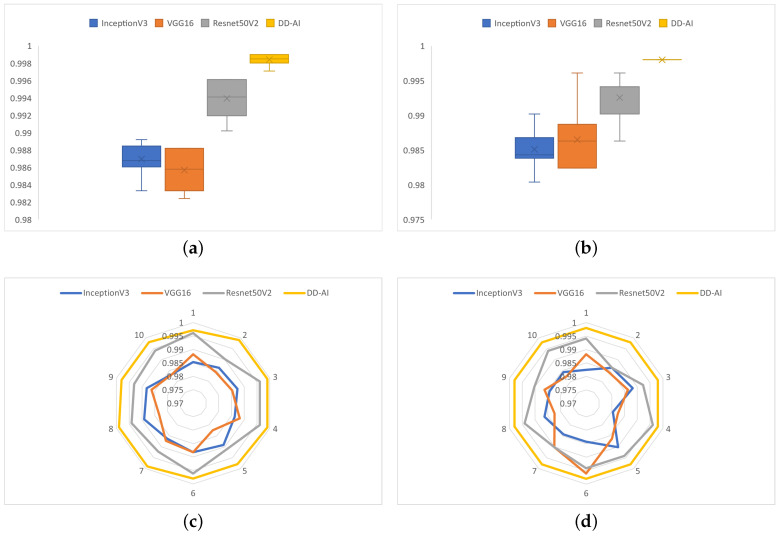
Performance and behavior of the models trained with the four CNNs: (**a**) boxplot of the accuracy, (**b**) boxplot of the recall for the drowsy class, (**c**) behavior on the ten experiments in terms of accuracy, and (**d**) behavior on the ten experiments in terms of recall of the drowsy class.

The performance and behavior of the models trained with the four CNNs are shown in Figure 16 in terms of the accuracy and recall metrics on the drowsy class. The DD-AI network performs best, with 99.84% average accuracy, followed by ResNet50V2 with 99.39% average accuracy, as shown in Figure 16a. Similarly, the DD-AI network performs better in average recall of the drowsy class, with 99.80% average recall, followed by ResNet50V2 with 99.25%, as shown in Figure 16b. In terms of behavior on the 10 training experiments, the DD-AI network presents more stable behavior of the accuracy metric compared to the other three networks, as shown in Figure 16c. Likewise, the DD-AI network presents completely stable behavior, with 99.80% recall of the drowsy class, whereas the other three networks present variable behavior in these ten training experiments, as shown in Figure 16d.

### 4.6. Step 6: Alarm Activation

As mentioned above, the proposed drowsiness detector system includes an audible alarm that is activated when the system detects the presence of drowsiness or yawning. The actuators are two speakers incorporated in the 7-inch display used to view the driver’s status in real time. When drowsiness or yawning is detected, it is displayed on the screen along with a drowsiness index and the level of mouth opening of the driver.

To activate the alarm through the two loudspeakers, the threshold for MAR must first be calculated using Equation (1), where an index of 55 was obtained with trial and error calculations. This indicates that if the MAR calculation is greater than 55, it is considered yawning; otherwise, it is a normal state. This index must be exceeded for at least 5 s. If these conditions are affirmative, an audible alarm is activated.

Similarly, the threshold of the drowsiness class must be calculated. If the model estimates that the probability of ROI extraction is higher than 95%, then it counts the time that the driver’s eyes remain closed. If this is greater than 300 ms [36], then a sound alarm is activated.

**Figure 17:**
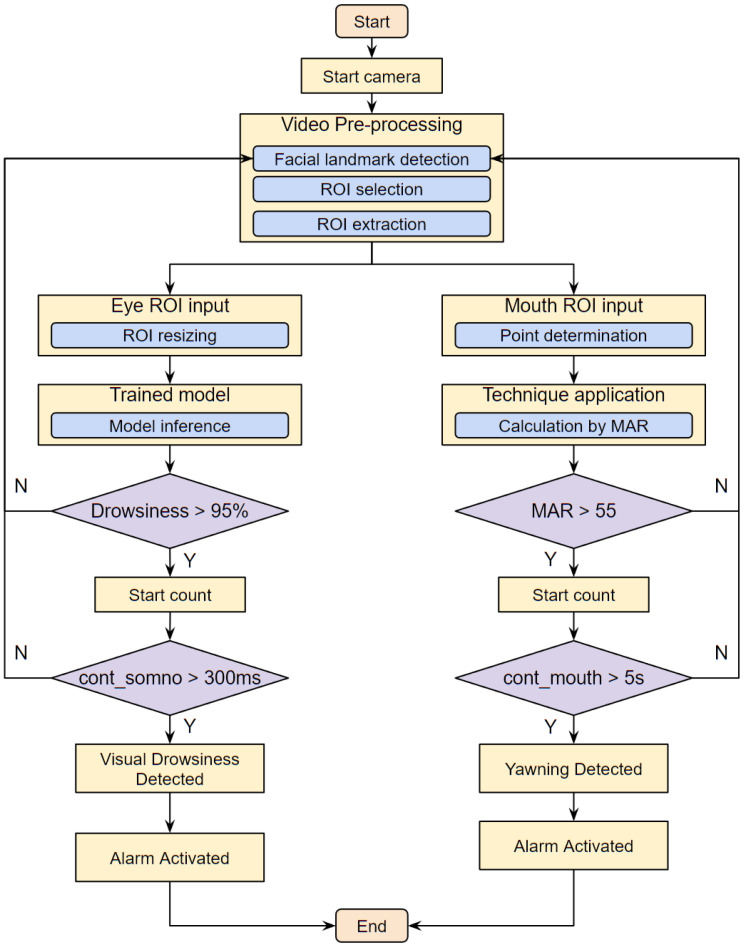
Drowsiness detection process flowchart.

A flowchart of the driver drowsiness detection process in a real environment using the mouth and eye states is shown in Figure 17.

## 5. Hardware Implementation

**Figure 18:**
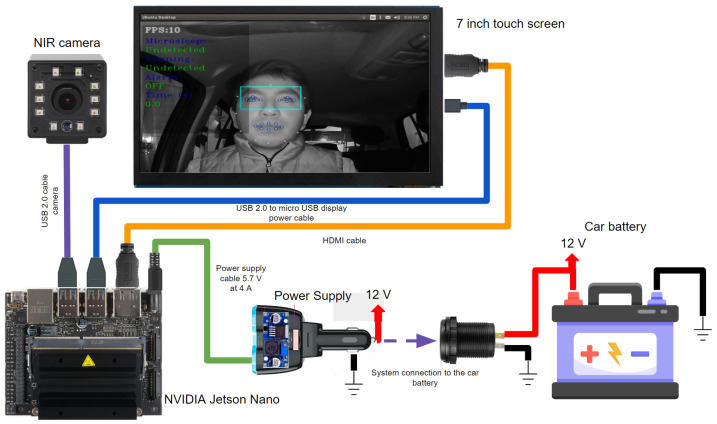
System connection diagram.

This section describes the hardware (embedded system) used to implement the algorithms of the four CNNs described in Section 4. In this particular case, there are several hardware options. The NVIDIA Jetson Nano was chosen as the hardware for implementation of the drowsiness detector system owing to its accuracy, time, memory, and power consumption with CNN algorithms according to the study by Süzen et al. [37]. The general architecture of the drowsiness detector system is presented in Figure 18. It is composed of four modules, which are described below.

The first module is the component that supplies power to the entire system through the embedded system; this module reduces the 12–24 V from the car battery to 5.5 V at 4A, which is the power needed for the hardware to work at its maximum performance. The second module is composed of an NVIDIA Jetson Nano embedded system, where program inference is performed.

The third module is composed of an NIR camera, which is in charge of receiving the driver’s video, focusing on the face and giving priority to the eyes and mouth. The last module is composed of a 7-inch touch screen with two built-in speakers for alarm activation. The driver can see whether he or she is drowsy or yawning through a graphical user interface (GUI), and alerts can be issued through the speakers.

**Figure 19:**
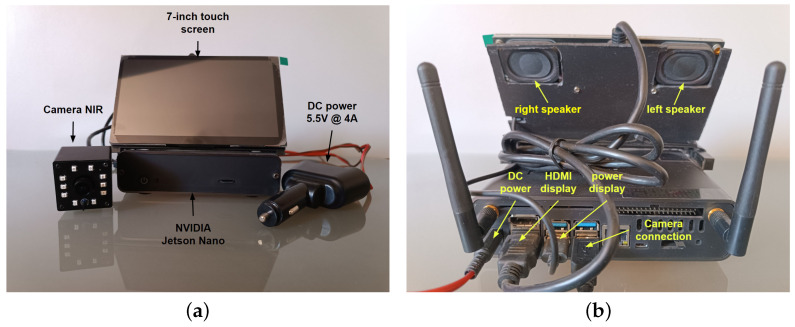
Installation of the drowsiness detection system: (**a**) front view and (**b**) back view.

The assembly of the drowsiness detector system is shown in Figure 19, showing the power supply and all of the components connected to the Jetson Nano, such as the NIR camera and display. It should be noted that the two speakers are located behind the display and come as one set. Figure 19a shows the four essential components of the system, while Figure 19b shows the connections of each component to the Jetson Nano.

## 6. Experimental Results

This section presents the experimental results. First, we present the performance of the software part of the system, where the results of the test part of each CNN network are shown. Next, we present the results of the implementation of the drowsiness detection system in hardware installed in a vehicle unit.

### 6.1. CNN Testing Results

**Figure 20:**
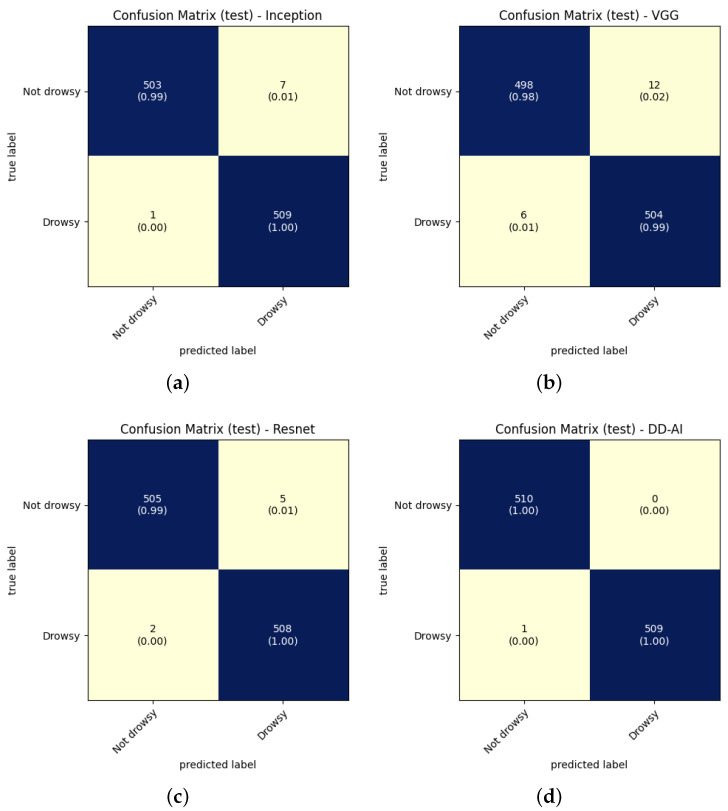
Confusion matrices from CNN tests: (**a**) InceptionV3, (**b**) VGG16, (**c**) ResNet50V2, (**d**) proposed “DD-AI”.

When training the models of the four CNN networks, testing must be performed with new images that the models have not yet seen. We proceeded to test each of the four trained networks ten times using the test folder. The same processing as in Figure 10 is applied here for the images that are inside of this folder while omitting data augmentation, image normalization, and resizing. The confusion matrices of each CNN network in the test set are shown in Figure 20.

The results on the test set are shown in Table 5 for the four evaluation metrics obtained from the confusion matrices of each of the four proposed networks. Equations (2)–(5) were used to obtain the evaluation metrics.

Analyzing the results in Table 5, it can be seen that our proposed DD-AI network has an average accuracy of 99.88% ± 0.1%, surpassing the ResNet50V2, InceptionV3, and VGG16 networks, which achieve average accuracy of 99.49% ± 0.3%, 98.95% ± 0.2%, and 98.33% ± 0.3% respectively. Looking at the recall metric on the drowsy class, our DD-AI network achieves 99.88% ± 0.1% on average, outperforming the other three networks.

**Figure 21:**
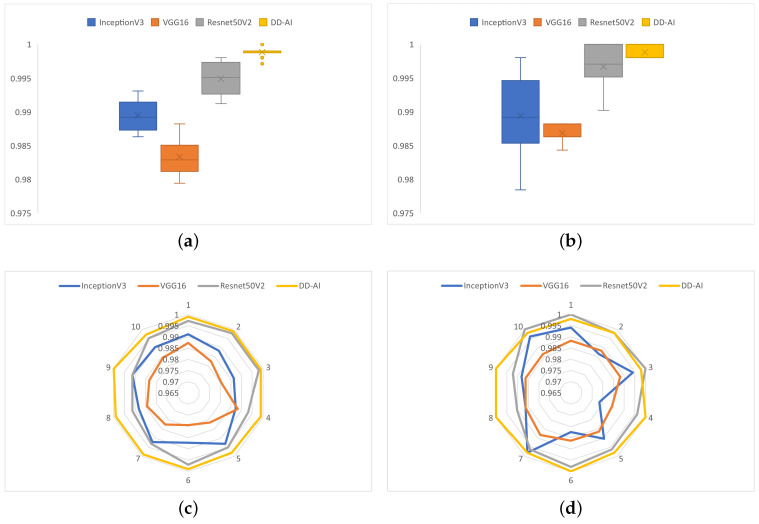
Performance and behavior of the test models of the four CNNs: (**a**) boxplot of the accuracy, (**b**) boxplot of the recall for the drowsy class, (**c**) behavior of the ten experiments in terms of accuracy, and (**d**) behavior of the ten experiments in terms of recall on the drowsy class.

Figure 21 shows the performance and behavior of each of the four networks on the ten test experiments for the accuracy and recall metrics on the drowsy class. The DD-AI network presents better performance in terms of the accuracy metric, with 99.90% average accuracy, and has similar results for the recall metric on the drowsy class, with 99.80% average recall, as shown in Figure 21a and Figure 21b, respectively. Likewise, the proposed DD-AI network presents more stable behavior in the case of the accuracy metric and the recall metric on the drowsy class, as shown in Figure 21c and Figure 21d, respectively. In comparison, the other three networks present variable behavior on the ten test experiments.

### 6.2. CNN Visual Result

**Figure 22:**
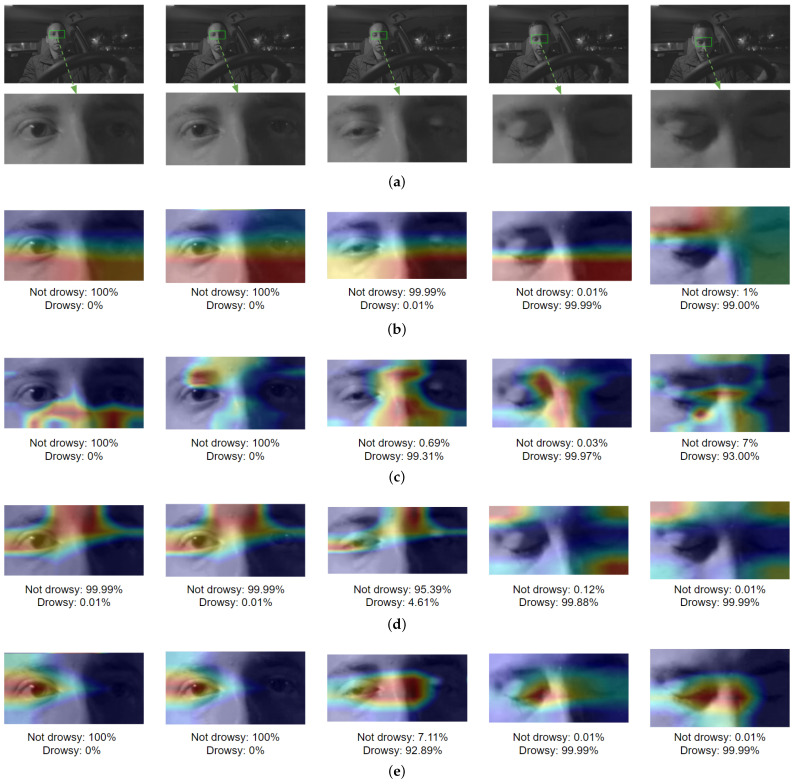
Grad-CAM visualization results of the four CNNs: (**a**) eye ROI, (**b**) InceptionV3 Grad-CAM, (**c**) VGG16 Grad-CAM, (**d**) ResNet50V2 Grad-CAM, (**e**) DD-AI Grad-CAM.

Another way to examine the four networks is to use the Grad-CAM [38] method, which is a visual explanation using heat maps to analyze which parts of the image led the CNNs to make their respective classification decisions. Five scenarios were chosen to represent the drowsiness sequence: the first two scenarios involved situations where the driver was not drowsy, in the third scenario the driver was entering the drowsy state, and in the last two scenarios the driver was drowsy. Figure 22 shows the Grad-CAM results for each of the four networks, where the weights of the networks were randomly chosen for visualization. Figure 22a presents the Grad-CAM evaluation of the ROIs extracted from the eye region and used for classification of sleepiness. As shown in Figure 22b, the InceptionV3 network primarily used the region under the eyes in the first four scenarios and the forehead region in the last scenario. The VGG16 network heat map in Figure 22c shows different regions of the ROI. On the other hand, Figure 22d shows that the the eyes and central part of the forehead are primarily used by the ResNet50V2 network in the first three cases, while in the last two scenarios the areas outside the eye region are dominant. Finally, the heat map for the proposed DD-AI network is shown in Figure 22e. It can be seen that the DD-AI network uses the eye region to determine whether the driver is drowsy or not in the five scenarios. demonstrating that the proposed network relies more on see the state of the eye area compared to the other three CNNs.

### 6.3. Overall Results of the CNNs

In addition, we assessed the total training time, the total weight of the training files of the four networks, the total number of parameters, and the response time of each network. All of these results are shown in Table 6.

### 6.4. System Performance Results

**Figure 23:**
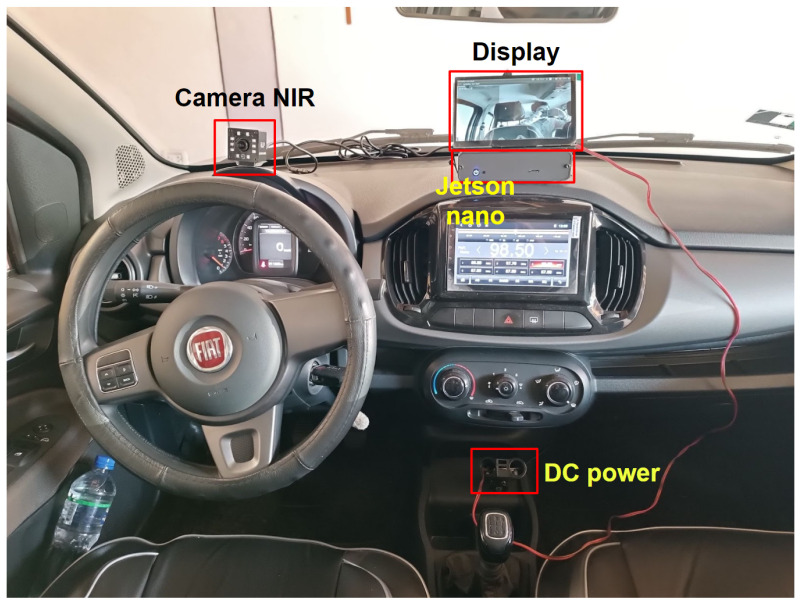
System installation in the vehicle.

**Figure 24:**
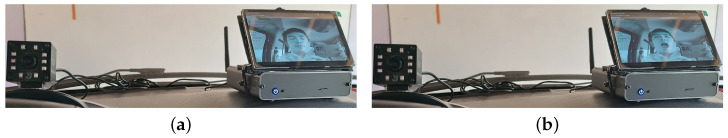
System functionality in two states of drowsiness: (**a**) microsleep and (**b**) yawning.

**Figure 25:**
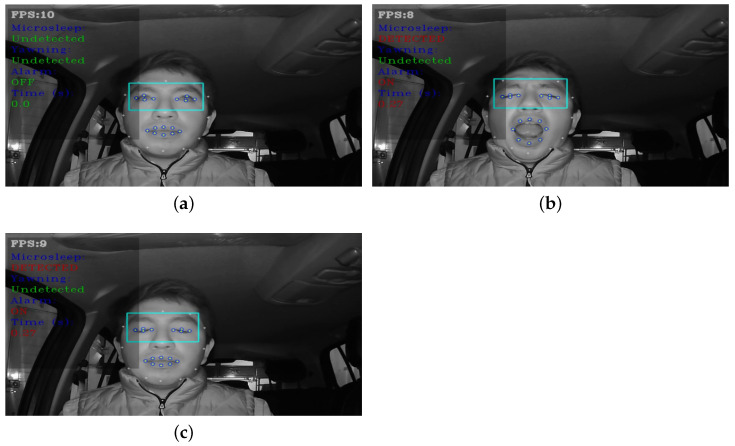
System operation of the GUI: (**a**) normal state, (**b**) yawning state, and (**c**) drowsy state.

This section present the results of the operation of the drowsiness detection system in a vehicle unit. The installation of the system inside the vehicle is illustrated in Figure 23. Figure 24 shows the operation of the system when the driver presents the two symptoms of drowsiness: microsleep (Figure 24a) and yawning (Figure 24b). Finally, Figure 25 shows the graphic interface (GUI) of the system, where the data used to detect microsleep and yawns in the three states can be seen. Figure 25a shows the driver in the normal state, Figure 25b shows the driver yawning, and in Figure 25c the driver is drowsy. It should be noted that the camera operates in infrared mode at all times during system operation.

After installation of the system in the vehicle, each CNN network was tested to validate its operation and performance inside the Jetson Nano. One model was chosen for each CNN network: for the CNN based on InceptionV3, experiment 7 was chosen; for the CNN network based on VGG16, experiment 4 was chosen; for the CNN network based on ResNet50V2, experiment 3 was chosen; and for the proposed CNN network, experiment 8 was chosen. The choice of the models was based on the accuracy and recall of the test results shown in Figure 21c and Figure 21d, respectively.

The overall accuracy and fps were calculated for each CNN, with the results shown in Table 7. The proposed DD-AI network had the best performance, with 96.55% accuracy within the embedded system, followed by the ResNet50V2 network with an accuracy of 95.86%, InceptionV3 network with 92.45%, and finally the VGG16 network with an accuracy of 92.45%. All CNN networks achieved average fps from 9 to 14, which is due to the understanding of the models by passing them inside the embedded system, which is sufficient to determine the drowsiness state by the state of the eyes and mouth. As 10 fps would be the minimum necessary to adequately detect a blink [39], this fps range ensures that the system can effectively capture eye movements associated with drowsiness. For yawning, an accuracy of 100% was obtained, as yawning detection is provided by the MAR equation.

## 7. Comparison and Discussion

This section presents a comparison and discussion of the results obtained by evaluating different drowsiness detection systems on various hardware platforms. The systems were tested in terms of accuracy and frame rate (fps), providing crucial information for their implementation in various applications. A comparison of the hardware used by each author, the fps, and the accuracy of each system is presented in Table 8.

All accuracy values were between 81% and 98.1%. The proposed system achieved 96.55% accuracy in the tests. The system in [19] presented the highest accuracy of 98.1%, although the microcontroller used was not specified. One aspect to consider is that their system issues an alert when the driver keeps their eyes closed for 5 to 8 s. This latency in the response makes it an inefficient system, as an accident could occur in that time. The system in [17] reached an accuracy of 97.44%, with the Eye Aspect Ratio (EAR) and Mouth Aspect Ratio (MAR) both used within the NVIDIA Jetson Nano hardware. The hardware used by [15,16,18], involving Convolutional Neural Networks (CNN) in their systems, obtained 94.05%, 89.55%, and 95.77% accuracy with 6 fps, 58 fps, and 21 fps, respectively, which represents a solid balance between speed and assumption in drowsiness detection. On the other hand, the hardware used by [12,13] was an Android cell phone. The authors used the CNN on both devices, achieving 83.3% accuracy with 234.25 fps on a Samsung Galaxy S8 Plus. This fps rate is too high for a device with limited features, considering that the Samsung Galaxy S8 Plus camera achieves fps from 30 to 60. The hardware used in [14] was a Raspberry Pi 4, with the CNN achieving 94.7% accuracy with 10.4 fps. This represents an attractive option due to the good balance between speed and accuracy and the affordable price of the Raspberry Pi. The NVIDIA Jetson TK1 hardware used by [11] presented very good results, with 89.5% accuracy at 14.9 fps, taking into account that the Jetson TK1 hardware was one of the first NVIDIA embedded systems.

The proposed system consists of Jetson Nano hardware with a 30 fps NIR camera. The use of MediaPipe reduces this to 20 fps, and an fps between 9 and 14 was obtained when using the CNN. Compared with the four other authors who made use of the Jetson Nano [15,16,17,18], our proposed system has better average fps performance compared to the 58 fps achieved by [16]; however, in the video provided by [18] it can be observed that the fps achieved by their system is less than 21 fps, while the system of [15] has a range of fps with the hardware. Our proposed system outperforms the four in accuracy by 96.55%, excluding the system in [17] that makes use of only MAR and EAR for drowsiness detection using the mouth and eye states and whose CNN network is designed only to detect whether or not the driver is wearing a mask. In addition to employing an Arduino Uno, AD8232 sensor, and other components, the total price of such a system amounts to approximately USD 185; in contrast, the system proposed in the present research has an estimated cost of USD 120, making it a more economical and accessible option for implementation without compromising functionality and effectiveness. A test run of the system can be downloaded from the Supplementary Materials.

The system proposed in this research demonstrates remarkable performance, with an accuracy of 96.55% and frame rate of 9 to 14 fps using Jetson Nano hardware and an NIR camera. Compared with similar systems such as [15,16,17,18], our method excels in accuracy and fps performance. However, its universality and applicability have some shortcomings. While the system strikes a balance between accuracy and cost, being more economical than other systems such as that of [17] that employ additional components, it still faces limitations in certain scenarios. A key challenge is the reduction in fps due to the computational cost of MediaPipe. In addition, although the system is competitive in terms of accuracy, this may vary depending on driver characteristics such as the use of facial accessories (masks) or when the face is not fully visible. Thus, additional adjustments in the training of the neural network may be necessary in order to improve its generalization capability. As seen in the Supplementary Materials, the system has a fast response in detecting drowsiness under various lighting conditions by analyzing the state of the driver’s eyes and mouth. This makes it more efficient compared to related work, improving its adaptability and accuracy in varying environments.

## 8. Conclusions and Future Works

This research presents a drowsiness detection system that combines the evaluation of the mouth state using the MAR metric and the use of a CNN to analyze eye drowsiness. We tested four CNN architectures: three based on InceptionV3, VGG16, and ResNet50V2, modifying the fully connected network architecture used in the classification process, as well as the proposed CNN network, which we name DD-AI. In addition, a new enhancement method is proposed to perform region of interest (ROI) extraction on the area surrounding the eyes.

Ten training experiments were performed for the InceptionV3, VGG16, ResNet50V2, and DD-AI CNN architectures. The proposed approach showed exceptional results in detecting drowsiness around the eyes, with accuracy values of 98.95%, 98.33%, 99.49%, and 99.88% respectively. In addition, the Grad-CAM technique was used to observe the behavior of each CNN at the time of inference to classify the state of the eyes. The proposed DD-AI CNN network presented a better performance with improved focus on the region of the eyes.

Subsequently, the system was implemented on Jetson Nano hardware and tested in a real environment by installing the system in a vehicle. The overall accuracy results were 92.45%, 90.27%, 95.86%, and 96.55% for the InceptionV3, VGG16, ResNet50V2, and DD-AI CNNs, respectively. The hardware test results showed slightly lower performance than the test results; however, all CNN architectures proved to be effective in detecting drowsiness in real driving conditions.

It should be noted that the proposed DD-AI CNN network performed the best in our hardware tests, with 96.55% accuracy and average fps between 9 and 14. This suggests that DD-AI is the most suitable architecture for deployment in real driving environments and can play a crucial role in preventing drowsy driving-related accidents. Overall, this system offers an effective and promising solution for addressing the problem of drowsy driving and improving road safety.

In future work, we intend to include driver distraction detection using head pose estimation. In addition, other types of hardware could be used for implementation. Additionally, we intend to make use of the IoT for constant monitoring of the driver for better centralized management of the system.

## Figures and Tables

**Table 1:** Factors involved in road accidents in Perú. Report by ONSV.

Factor	2019	2020	2021	2022
Human factor	75.5%	73.8%	69.1%	70.4%
Vehicle factor	2.1%	2.0%	2.0%	1.7%
Infrastructure factor	3.3%	2.7%	3.2%	2.9%
Other factors	19.1%	21.5%	25.8%	24.9%

**Table 2:** Dataset Distribution.

Data Set	Drowsy	Not Drowsy
Training Set	2380	2380
Validation Set	510	510
Test Set	510	510

**Table 3:** Training parameters.

Hyper-Parameters	Value
Optimizer	ADAM
$\beta_{1}$	0.001
$\beta_{2}$	0.9
Learning rate	0.999
Epochs	30
Batch size	32
Number of experiments	10 for each CNN

**Table 4:** Evaluation metrics on the validation set.

Model	Class Name	Precision	Recall	F1-Score	Accuracy
Inception V3	Not drowsy	0.9852 ± 0.003	0.9888 ± 0.003	0.9870 ± 0.002	0.9870 ± 0.002
	Drowsy	0.9888 ± 0.003	0.9851 ± 0.003	0.9869 ± 0.002	
VGG16	Not drowsy	0.9865 ± 0.004	0.9849 ± 0.004	0.9857 ± 0.002	0.9857 ± 0.002
	Drowsy	0.9849 ± 0.004	0.9865 ± 0.004	0.9857 ± 0.002	
ResNet50V2	Not drowsy	0.9926 ± 0.003	0.9953 ± 0.003	0.9939 ± 0.002	0.9939 ± 0.002
	Drowsy	0.9953 ± 0.003	0.9926 ± 0.003	0.9939 ± 0.002	
Proposed DD-AI	Not drowsy	0.9980 ± 0.000	0.9988 ± 0.001	0.9984 ± 0.001	0.9984 ± 0.001
	Drowsy	0.9988 ± 0.001	0.9980 ± 0.000	0.9984 ± 0.001	

**Table 5:** Evaluation metrics on the test set.

Model	Class Name	Precision	Recall	F1-Score	Accuracy
Inception V3	Not drowsy	0.9895 ± 0.006	0.9896 ± 0.006	0.9895 ± 0.002	0.9895 ± 0.002
	Drowsy	0.9897 ± 0.005	0.9894 ± 0.006	0.9895 ± 0.002	
VGG16	Not drowsy	0.9868 ± 0.001	0.9798 ± 0.006	0.9833 ± 0.003	0.9833 ± 0.003
	Drowsy	0.9800 ± 0.006	0.9869 ± 0.001	0.9834 ± 0.003	
ResNet50V2	Not drowsy	0.9967 ± 0.003	0.9931 ± 0.003	0.9949 ± 0.003	0.9949 ± 0.003
	Drowsy	0.9932 ± 0.003	0.9967 ± 0.003	0.9949 ± 0.003	
Proposed DD-AI	Not drowsy	0.9988 ± 0.001	0.9988 ± 0.001	0.9988 ± 0.001	0.9988 ± 0.001
	Drowsy	0.9988 ± 0.001	0.9988 ± 0.001	0.9988 ± 0.001	

**Table 6:** Evaluation Metrics on the test set.

Model		Result in Training File Size (KB)		Result in Testing Response Time
	Training Time		Training Time	
InceptionV3	4.3 min ± 2 s	98,055	22,828,286	51.01 ms
VGG16	3.1 min ± 3 s	69,586	15,740,190	39.00 ms
ResNet50V2	3.4 min ± 3 s	140,594	27,662,302	60.02 ms
Proposed DD-AI	3.1 min ± 1 s	75,618	6,448,002	33.00 ms

**Table 7:** Overall hardware performance.

CNN	Accuracy	FPS
InceptionV3	92.45%	9–14
VGG16	90.27%	9–14
ResNet50V2	95.86%	9–14
DD-AI	96.55%	9–14

**Table 8:** Comparison of drowsiness detection systems.

System	Used Hardware	FPS	Accuracy
Reddy et al. [11]	NVIDIA Jetson TK1	14.9	89.5%
Jabbar et al. [12]	Android Phone	-	81%
Jabbar et al. [13]	Samsung Galaxy S8 Plus	234.25	83.3%
He et al. [14]	Raspberry Pi 4	10.4	94.7%
Çivik and Yüzgeç [15]	NVIDIA Jetson Nano	6	94.05%
Li et al. [16]	NVIDIA Jetson Nano	58	89.55%
Rahman et al. [17]	NVIDIA Jetson Nano	-	97.44%
Flores-Monroy et al. [18]	NVIDIA Jetson Nano	21	95.77%
Singh, N.T. et al. [19]	unspecified	-	98.1%
Proposed DD-AI	NVIDIA Jetson Nano	14	96.55%

## Data Availability

The public database used in this paper was NITYMED (Night-Time Yawning–Microsleep–Eyeblink–Driver Distraction), which can be found at https://datasets.esdalab.ece.uop.gr/ (accessed on 2 November 2022).

## Supplementary Materials

The following supporting information can be downloaded at: https://www.mdpi.com/article/10.3390/s24196261/s1, Video: test-system.mp4.
